# Navigating facilitated regulatory pathways during a disease X pandemic

**DOI:** 10.1038/s41541-020-00249-5

**Published:** 2020-10-23

**Authors:** Shmona Simpson, Ajoy Chakrabarti, David Robinson, Keith Chirgwin, Murray Lumpkin

**Affiliations:** The Bill and Melinda Gates Foundation, 500 5th Ave N, Seattle, WA 98109 USA

**Keywords:** Drug regulation, Drug safety, DNA vaccines, Gene therapy, Recombinant vaccine

## Abstract

In 2018, the Bill and Melinda Gates Foundation convened over thirty subject matter experts in clinical development, manufacturing, and regulatory assessment to determine how the development and approval of medical countermeasures could be accelerated in the event of Disease X. Disease X is the result of a presently unknown pathogen with epidemic or pandemic potential. A key opportunity to accelerate the scientific assessment and regulatory approval of medical countermeasures exists within efficient navigation of facilitated regulatory pathways. It was identified that not all stakeholders will be able to skillfully navigate the facilitated pathways offered by the various regulatory agencies during a public health emergency. To democratize this knowledge, we have written an overview of the facilitated approaches which have been developed and refined by Stringent Regulatory Authorities and the World Health Organization for the primary assessment of medical products. We discuss the conditions necessary for use of these approaches, scenarios in which certain pathways may be applicable, and the pros and cons of these approaches. We also address opportunities available to developers in, or developers who wish to access, low-income countries that may have nascent regulatory frameworks.

## Introduction

The 2014–2016 Ebola epidemic significantly impacted the global health community. Between Guinea’s index case in December 2013 and the epidemic’s end in June 2016, there were 28,000 cases and 11,325 deaths across eight countries^1^. Despite years of prior research, no products were ready to deploy in time to save these lives. The question then arose: How many lives would have been saved if effective medical countermeasures had been made available sooner?

The WHO created the Research and Development Blueprint initiative^2^, which asked multiple agencies how to shorten the time to development of medical countermeasures for the world’s most deadly pathogens. The Bill and Melinda Gates Foundation attempted to answer this question for Disease X—the result of a presently unknown pathogen with epidemic or pandemic potential. In December 2018, the Foundation convened a Disease X working group—comprised of 30 experts in emerging infectious diseases; bioterrorism agents; non-clinical studies; clinical trials; chemistry, manufacturing, and controls; and regulatory scientific assessment^3^. The goal was to understand how the next epidemic could be controlled using medical countermeasures that arrive sooner than has been the experience to date. The control of Disease X requires improvements in many areas, including disease surveillance, public health infrastructure, laboratory capacity, product manufacturing, and delivery^4^—however the remit of this working group was to focus specifically on the expedited provision of medical countermeasures. One of the key challenges identified was a perceived difficulty in navigating facilitated regulatory pathways.

In this Perspective, we summarize some facilitated regulatory pathways available to innovators tackling Disease X and suggest how these may shorten product development and regulatory assessment timelines. We also discuss the pros and cons of these approaches and suggest in which situations they may be most applicable. So far, we have identified 50 facilitated regulatory pathways (listed in Supplementary Note 1), in 24 countries around the world. While many of these have critical nuances and are at various stages of refinement^5,6^, they cannot all be adequately described in this short Perspective. A broader set of considerations are available elsewhere^6,7^. Rather, some “Stringent Regulatory Authorities” (as classified by WHO) have completed primary assessment of thousands of products using these facilitated approaches. These include products used in epidemic emergencies. Many other National Regulatory Agencies (NRAs) have mirrored these processes, and/or rely on the scientific outcomes of these approaches to assist in their own authorization practices.

This Perspective provides a high-level overview of the facilitated approaches which have been developed and refined by Stringent Regulatory Authorities and the World Health Organization for the primary assessment of medical products. It is possible that these represent the approaches most likely to be relied upon in the next epidemic or pandemic. We hope this paper will enable academic innovators and small and medium enterprises to navigate the flexibility that exists within regulatory approaches for products that address life-threatening diseases of unmet need. We encourage developers to make early contact with regulators in their focus countries to discuss which programs may be applicable.

## When are facilitated regulatory pathways warranted?

Several potential regulatory scenarios may exist and co-exist during an epidemic: for example, (a) de-novo candidates requiring rapid development and regulatory assessment (b) de-novo products requiring assessment when the typical package of clinical efficacy data may not be available, (c) approval of de novo or repurposed products for “emergency” use only in specific populations (d) for compassionate use in specific (e.g., “named”) individuals of an unauthorized medicine (e) conditional or accelerated authorization before the completion of efficacy studies or, (f) use of a licensed product outside of its approved use (e.g., for another indication, dosage regimen, or population).

Many regulatory agencies have instituted various programs to help navigate these scenarios (Table 1).

**Table 1:** Facilitated regulatory approaches.

Agency	Name of initiative	Basic requirements	Regulatory scenario
United States food and drug administration (FDA)	Emergency use authorization (EUA)^11^	•Declaration of US health emergency •Early human safety data •Strong plausibility of human efficacy	Public health emergency where high disease risk makes demonstrating efficacy challenging
	Animal rule (AR)^12^	•Strong animal efficacy data in two models and some human clinical safety data •Human efficacy trials not feasible/ethical	Significant public health need where human efficacy cannot be ethically demonstrated
	Expanded access (EA)^13^	•Generally, complete clinical trial safety and efficacy data; individual patient use may require less •Product generally must be awaiting •assessment for marketing authorization	Bridging access between clinical trials and authorization for emergency or compassionate use in named patients
	Accelerated approval^14^	•Well controlled studies that demonstrate an effect on an unvalidated surrogate or intermediate endpoint •Studies continue post-authorization	Serious disease without satisfactory therapy. Rapid assessment without typical efficacy package
	Priority review^15^	•Putative evidence of superior effectiveness or safety over an available therapy	Rapid assessment, decision received within 6 months
	Fast track^16^	•Early putative evidence of superior effectiveness or safety over an available therapy–also if there is no current therapy	Serious/life-threatening disease. Facilitated assessment through rolling review process
	Breakthrough therapy designation^17^	•Pharmacodynamic biomarker, surrogate or intermediate clinical endpoints that strongly suggest an improved clinical effect when compared to an available therapy or if there is no current therapy	Serious disease without satisfactory therapy. Rapid assessment based on early clinical data. Opportunity for frequent regulatory interaction.
World Health Organization (WHO)	Emergency use listing procedure (EUL)^18^	•Strong plausibility of human efficacy and safety •Early human safety data •Some clinical safety data where possible	Public health emergency allowing listing of product based on benefit/risk. Approval could be granted within 90 days
European medicines agency (EMA)	Accelerated assessment^19^	•Candidates with sufficient pre-clinical and clinical data are eligible for accelerated assessment reducing assessment time from 210 to 150 days	Serious disease without satisfactory therapy, or where therapy can be improved. Rapid assessment
	Conditional marketing authorization (CMA)^20^	•Strong plausibility of human efficacy •Early human safety data •Studies continue post-authorization	Serious or life-threatening disease without satisfactory therapy. Rapid assessment without typical efficacy package showing clinical benefit at time of authorization
	Marketing under exceptional circumstances^21^	•Plausibility of human efficacy •Possibly no clinical data requirement providing the benefits significantly outweigh costs	Significant public health need where human efficacy cannot routinely be demonstrated
	Priority medicines with accelerated assessment scientific advice and protocol assistance (PRIME)	•A strongly substantiated mechanism of action, preclinical data, and human tolerance data •Academic, small and medium enterprises may apply earlier for advice and protocol assistance	Serious or life-threatening condition for which therapy is inadequate. Rapid assessment, based on early clinical data
Japan Pharmaceuticals and medical devices agency (PDMA)	Priority review^22^	•Available for orphan designated products, and those that receive conditional approval for diseases of unmet clinical need	Rapid Assessment, decision received within 9 months
	Conditional term-limited approval^22^ (Regenerative Products)	•Promising phase I/II efficacy and safety data •Must conduct post marketing confirmatory studies within 7 years–but randomized studies are not necessary	Serious or life-threatening disease without satisfactory therapy. Rapid assessment without typical efficacy package showing clinical benefit at time of authorization
	Conditional approval^23^	•Exploratory clinical studies which show safety and efficacy, but confirmatory studies are not required	Serious or life-threatening disease without satisfactory therapy. Rapid assessment without typical efficacy package showing clinical benefit at time of authorization
	Sakigake (Pioneer)^24^	•Product for serious disease with unmet medical need •Intention to File in Japan •Non-clinical efficacy, early clinical data, and a mechanism of action which suggests efficacy	Serious or life-threatening disease without satisfactory therapy. Rapid assessment without typical efficacy package showing clinical benefit at time of authorization. Decision within 6 months

**Fig. 1:**
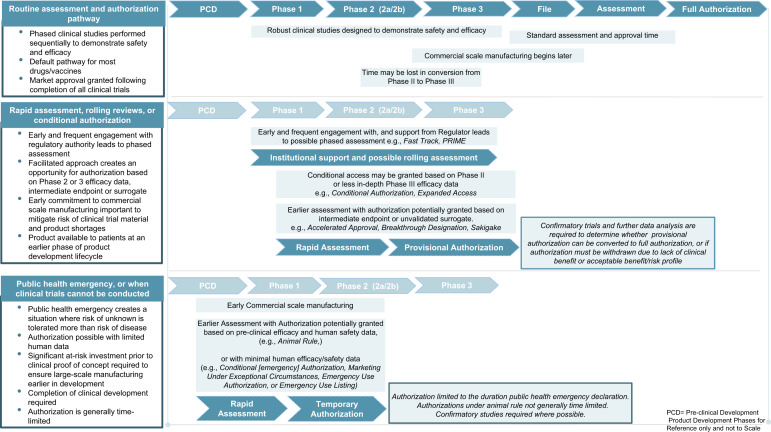
Navigating facilitated regulatory pathways during an epidemic or pandemic. Top. In the routine assessment and authorization pathway, market authorization is granted following the completion of successful phased clinical trials, analysis of comprehensive data sets from adequate and well-controlled trials, and a standard regulatory assessment timeline. Middle. Rapid assessment and authorization based on less comprehensive data derived earlier in product development shorten the overall timeline for serious indications of unmet clinical need. Rolling reviews, increased institutional support, and frequent scientific advice may accelerate the development and assessment processes. In some jurisdictions, an authorization with conditions to be met post-authorization may be granted–bridging the gap between when definitive clinical trials are conducted and market authorization. In some cases, intermediate endpoints or unvalidated surrogates may be sufficient for an initial conditional or accelerated authorization to meet unmet clinical needs for serious and life-threatening diseases. Further confirmatory studies are required post-authorization to either validate the clinical benefit assumptions or not. If not validated, the product is removed. These pathways run the risk of possibly depleting clinical trial material potentially delaying larger-scale trials. Another risk relates to not having adequate capacity to supply need if investments in manufacturing capacity are not made earlier enough in product development. Bottom. In declared public health emergencies, temporary authorization may be given based on promising pre-clinical data and early human clinical (if possible) and safety data. Such authorizations are usually valid for the duration of the declared emergency. These approaches require significant at-risk investment, ensuring large scale manufacturing early on, in order to meet the need of the emergency. The animal rule is an option in both routine and emergency situations. It allows authorization based on animal efficacy and human safety data, when it is not ethical or possible to conduct human efficacy trials.

These pathways are generally reserved for products that address a serious or life-threatening condition where there is unmet clinical need, or where the current treatment options are unsatisfactory. Eventual authorization through one of these pathways depends on an evaluation of the known clinical benefits and risks of the product—in the context of the known risks of the disease. For this, regulators employ a variety of complex modeling and analytic techniques to conduct an assessment of the benefits and risks of the product and any remaining uncertainties, and compare these to the risks of the disease and the available options at the time of application^8,9^. In an outbreak, these parameters can be highly variable; they may change depending on the pathogen, co-morbidities, evolution of the epidemic, populations and geographies affected. However, these agile facilitated regulatory pathways recognize the higher tolerance for unknown risk the community has in these situations: they allow for flexibility on the depth of certain routine data requirements given the specifics of the disease being considered. Not all of these pathways facilitate a shorter assessment time: some allow for assessment to be conducted at an earlier phase of the typical product development lifecycle in instances where the benefits, at that point in time, outweigh the risks (Fig 1).

A critical element for successful use of these facilitated pathways is the engagement of regulators early and often: scientific advice and pre-submission meetings are invaluable. Most Authorities allow rolling submissions of data and rolling reviews under these processes. This early engagement allows for on-going alignment on development plans as further data become available. It also allows sponsors to focus on the critical data requirements, identify opportunities for additional product development acceleration, and ultimately save substantial time. Depending on the product and the clinical situation, a product may be eligible for any or all these facilitated programs and can be candidates in more than one program simultaneously. Without active regulator engagement, it is often difficult to navigate these approaches and understand the challenges inherent in each.

Regulation through reliance and regionalization are critical elements for broadening the utility of facilitated pathways. Reliance allows for NRAs to rely on the work product of a trusted Authority to inform their own regulatory decision. Regionalization extends the utility, allowing neighboring regulatory authorities to workshare, and share work product, within their economic or cultural blocs. This may mean that regulatory assessment and authorization in one jurisdiction, can de facto facilitate accelerated authorization in another jurisdiction thereby avoiding duplication of effort^10^. A list of regulatory harmonization initiatives is provided in the Supplementary Note 1.

## Facilitated regulatory approaches in routine and emergency situations

*The following pathways are available under certain circumstances to expedite product development and marketing application assessment*.

*The United States Food and Drug Administration’s (FDA) Priority Review*^11^ process provides feedback on a marketing application, i.e., an authorization or complete response, within, generally, 6 months, instead of the standard 10 months. This faster application review is for products that purport to demonstrate significant efficacy or safety over a currently available therapy related to the treatment, diagnosis, or prevention of a serious condition. This designation, which comes at the time of submission, does not affect the length of the clinical trial period. Many other regulatory authorities have expedited review timeframes for similar situations, and one should always check to see if the medical countermeasure would qualify in the country in which the product is intended to be used. Accelerated assessment, under similar situations, is also available at the European Medicines Agency (EMA), reducing review time from 210 days to 150 days.

*Japan’s Pharmaceutical and Medical Devices Agency (PMDA)* also provides a *Priority Review* option, available to products that address (1) serious diseases, (2) conditions of unmet clinical need, or where superior safety and efficacy can be provided, and (3) orphan designated products. This option reduces review time from 12 to 9 months^12,13^.

*FDA’s Fast Track* is a program for products that have some initial evidence of efficacy or improved safety over an available therapy^14^. This designation provides an opportunity for frequent meetings and communication with the FDA. Most importantly, this program allows a “rolling review” process, in which the marketing application is submitted in pieces as each segment is completed rather than having to assemble the entire application and submit it all at once. This allows FDA to assess each segment as it is submitted, and thus FDA only must review the last segment when it is completed rather than the entire application.

*FDA’s Breakthrough Designation*^15^ is granted where a new candidate demonstrates substantial improvement over an available therapy on a clinically significant endpoint. Candidates demonstrating an improved safety profile over an available therapy with similar efficacy are also considered. Efficacy can be demonstrated using a pharmacodynamic biomarker, surrogate or intermediate clinical endpoint providing they strongly suggest a clinically meaningful effect. For example, Pfizer’s 20-Valent Pneumococcal Conjugate Vaccine candidate and Janssen’s prophylactic vaccine for the prevention of respiratory syncytial virus both achieved breakthrough designation following a Phase 2 and 2b studies, respectively^16,17^. A breakthrough therapy designation enables fast-track designation, intensive guidance on a drug development program, and organizational commitment.

Like FDA’s Breakthrough program, investigational products to address unmet medical need are eligible for consideration under the European Union’s *PRIority MEdicines (PRIME)* scheme, providing early clinical data demonstrate potential benefit. In addition, applicants from the academic sector, small and medium enterprises can engage with the EMA quite early based on compelling non-clinical data and tolerability data from initial clinical trials. Not exclusive to these applicants, the EMA also offers scientific advice and protocol assistance to ensure the most expeditious experience with applying for market authorization^18^. PRIME also ties in to accelerated assessment if the data ultimately demonstrate the level of improvement needed for accelerated assessment designation.

*The following pathways are reserved for candidates when the benefit risk analysis indicates that access should be granted even if the entire clinical trial process has not been completed. Generally, these are temporary authorization statuses and are not intended to replace or circumvent ultimately finalizing the clinical trials required to support full market authorization. As a condition of approval under these pathways, it is general a requisite that the clinical trials be continued until adequate clinical efficacy and safety either are or are not demonstrated*.

*FDA’s Accelerated Approval* allows for authorization where efficacy is demonstrated via an unvalidated surrogate endpoint or an intermediate clinical endpoint^19^. These surrogate endpoints are likely predictors of clinical benefit and require that this be demonstrated by “adequate and well controlled” studies. Ultimately, the requirement remains to confirm clinical benefit in post-authorization confirmatory trials that validate the approved endpoint. When these studies are completed, the FDA will review the data and decide if the approval can be converted to a full authorization. The accelerated approval may be revoked if clinical benefit relative to the risks cannot be confirmed. To date, 208 compounds have been approved under this pathway. These range from numerous antiretroviral compounds in the 1990’s, to Janssen’s Levaquin for aerosolized *Bacillus anthracis*, to the recent approval of Ismed’s Arikayce for treatment of mycobacterium complex in 2018—all averaging an initial accelerated approval time of six months^20^.

*FDA’s Expanded Access (EA)* is a program designed for patients with an immediately life-threatening disease to access a product that has clinical trial data (putatively showing an acceptable benefit-risk profile)—but does not yet have marketing authorization. Because the authorization has not yet been granted, the product is considered investigational and therefore written informed consent of the patient must be obtained when used. Generally, EA is used in situations where alternative therapies are not available. Which EA program is chosen reflects the perceived need for the product in terms of number of patients. Under such programs, the product can be used for: (1) a single patient, (2) immediate-size populations that occur after the FDA has received a number of requests for single patient use, or (3) under a “treatment investigational new drug,” designation. These are generally used during the time period after the completion of pivotal trials, but before the authorization is granted. During this period, there may be large numbers of patients that might benefit from the product during the time the marketing application is being assembled and/or the product is under review^21^. Options for international expanded access exist in 21 CFR 312.110(b)(ii)^22^ which allows for the export from the United States of investigational products for national emergencies elsewhere, with NRA approval in the receiving country. For example, following promising animal and early-phase clinical studies (PREVAIL II Trial) the FDA supported the use of ZMapp through a standing expanded access protocol prior to completion of the submission, which allowed countries to retain access to vital therapeutic agents^23^.

Manufacturers who provide access to product under one of the “expanded access” programs may only recover the direct costs of manufacturing their investigational product and may not recoup additional costs or make a profit. In these cases, access to product under an “expanded access” program allows for patients to receive the product when the potential benefit outweighs the known risk in the specific context of the patient(s). This sometimes provides further valuable data to help support a full marketing application.

The EMA also offers programs to bridge the clinical trial and full authorization gap. Unlike a typical full marketing authorization, a *Conditional Marketing Authorization (CMA)*^24^ can be granted in instances of unmet medical need where the benefit-risk assessment is positive. This is based on early data suggesting that the sponsor will be able to provide more complete data within an agreed timeframe to validate the tentative positive benefit-risk profile of the product. These data generally include more comprehensive clinical efficacy and safety data^25^. CMA’s are generally granted for a 12-month period, after which they are further reviewed in light on any new data available. If deemed of benefit to public health, the conditional authorization can be extended. A large number of antiretroviral compounds^26–28^ and treatment for multi-drug resistant tuberculosis such as Bedaquiline^29^ and Delamanid^30^ continue to be marketed conditionally by the EMA as trials are ongoing. Ultimately, this pathway this may provide a route to full authorization for many of these candidates.

These provisions, both in the US and EU, require post-authorization infrastructure so that further, more comprehensive data can be captured and so that any requisite safety monitoring can be performed. Such infrastructure often does not exist in low-income countries. This makes use of these pathways sometimes difficult, if not impossible, in countries without such needed infrastructure.

A key feature of these mechanisms is that they often feed into each other and are not meant to be mutually exclusive. For example, in 2018, 60 Degrees Pharmaceutical’s Tafenoquine for malaria prophylaxis was awarded both Fast Track and Priority Review designations^31^. Similarly, a medicine in the PRIME scheme could also be granted conditional marketing authorization during clinical trials, while still benefitting from accelerated assessment and eventual full authorization^32^.

*Japan’s PDMA* offers two conditional approval options; the first is for regenerative products which grants a term-limited approval based on exploratory Phase I and II safety and efficacy data^12^. Confirmatory studies are required post-market, and applications must be submitted for complete market authorization within 7 years. This is described as conditional “term limited” approval. The second conditional approval is applied in instances of a serious disease, disease of unmet clinical need, or where it is too difficult or excessively lengthy to conduct efficacy studies. In this instance, early phase clinical studies must show some safety and efficacy, and post-market requirements will include surveillance or clinical studies^33^.

Japan’s Sakigake (or pioneer) program is available for diseases of unmet clinical need where the sponsor agrees to file the marketing application first in Japan (or simultaneously first in Japan and another country). For a candidate to qualify for Sakigake, there must be some early phase clinical data and strong non-clinical and mechanism of action data. This program offers pre-application consultation, expedited review of around six months, and superior support from PDMA. The re-examination of clinical safety and efficacy data can be lengthened for an indeterminate period of time, in order to strengthen the likelihood of full market authorization^34^.

## When clinical trials are unethical or unfeasible in routine and emergency situations

When conducting clinical trials presents insurmountable logistical, safety, or ethical challenges, the following regulatory approaches allow for efficacy to be demonstrated using an animal surrogate or clinically indicative endpoints alongside human safety data. These are reserved for when public health need is significant and human efficacy testing is not possible. In all instances, every attempt is made to procure follow-up analyses of human experience with the product post-authorization.

The *FDA’s Animal Rule*^35^ provides a pathway to approve novel candidates in the absence of the demonstration of efficacy in clinical trials. Where clinical trials are impossible or unethical to conduct, determinations of efficacy are primarily based on studies in well-characterized animal models^36^. The investigator must provide:

- poof that the animal model can provide plausible inference for human efficacy,
- proof that the mechanism of action is the same in the model, as it would be in humans,
- rationale for the dose provided to humans (which may come from human pharmacokinetic studies and animal efficacy studies).

In these situations, safety is demonstrated using traditional toxicology studies and human experience data (usually from Phase 1 trials in healthy human volunteers). The chemistry, manufacturing, and controls information needed to support an animal rule application follow FDA guidelines for a routine new drug or biological license application. This program allows a promising candidate to be authorized for human use where a major public health need is justified. This authorization is rare: to date only 6 and 8 drug and biologic approvals, respectively, have been granted under this provision^37^. Built into the “animal rule” is an iterative process where post-authorization human safety and efficacy data can be obtained and used as the product is employed to further refine the product label. Aside from *de-novo* product authorization, the animal rule may also be used to gain authorization for a new indication. In addition to several medicines that have been approved under this provision, the anthrax vaccine BioThrax was approved in 2015 for post-exposure prophylaxis making it the first vaccine to receive approval for a new indication based on the Animal Rule^38^.

In the European Union, an analogous option exists within the provisions of the “*Marketing Under Exceptional Circumstances*” pathway. In exceptional circumstances, when an applicant cannot reasonably be expected to conduct clinical trials, they may submit as much clinical safety as efficacy data as possible, alongside a proposal for post-approval studies that would support the original safety and efficacy claims. Inability to supply a complete efficacy package may occur due to the rarity of the disease, limitations in the present state of scientific knowledge, or when it would be unethical to conduct. Once marketed under exceptional circumstances, the candidate will be supplied with a summary of product characteristics stating that information regarding the candidate is incomplete, and the label may be updated^39^. In 2013, the smallpox vaccine Imvanex was marketed under exceptional circumstances after exploratory data demonstrated that protective antibodies could be triggered, with only mild side effects (including in patients with HIV or atopic dermatitis). To date, the vaccine’s benefits and risks continue to be studied in vaccine recipients. Ultimately true efficacy could only be demonstrated if there is an outbreak of the disease in the future^40^.

## Special authorizations during public health emergencies

*The following pathways are reserved for when a national or global public health emergency has been formally declared*.

*FDA’s Emergency Use Authorization (EUA)* is enabled once the United States Secretary of Health and Human Services declares a specific national public health emergency^41^. It considers whether the “known and potential benefits of the product outweigh the known and potential risks” providing that no reasonable alternatives exist for treating the cause of the specified national public health emergency. Data to support the application may include domestic and/or foreign clinical trial data, in vivo animal data, and in vitro data that provide plausible support for clinical efficacy. Access to a product under an EUA is limited to the duration of the national health emergency and the specific access caveats imposed. Thereafter the product is again considered investigational. For example, during the 2009 H1N1 influenza outbreak, EUAs were granted for the dispensing of Tamiflu (oseltamivir phosphate) and Relenza (zanamivir), and intravenous Peramivir. These were discontinued in 2010 once H1N1 was no longer deemed a threat to the United States^42^. This pathway is applicable to medicines, biologics, vaccines, and medical devices, including in vitro diagnostics. So far, the EUA has been utilized primarily for diagnostic tests for several infectious agents including influenza, anthrax, coronaviruses, ebolavirus, and zika viruses.

For those developing products to be used in countries with nascent or under-resourced regulatory agencies, the WHO has provided the *Emergency Use Listing (EUL)* process to provide guidance on product quality and use during a public health emergency of international concern (PHEIC) or other specified public health emergency. Only the Director General of the WHO can authorize the use of the EUL process. This program was developed and first used for Ebola diagnostics during the 2013–2015 West African Ebola outbreak and was previously referred to as the Emergency Use Assessment and Listing (EUAL) process. Since then, it has been further refined and renamed, with the most recent guidance issued in early 2020^43^.

This process was used extensively in both the Ebola and Zika outbreaks for in vitro diagnostic products, although the process is clearly intended for medicines and vaccines also. The first example of a vaccine advancing into the EUL process is the new oral polio vaccine for type 2 virus (nOPV2). This genetically modified (designed to improve safety relative to the existing oral polio vaccine, mOPV2), oral polio vaccine is being developed by Bio Farma (Indonesia) and PATH. On-going transmission of type 2 Vaccine-Derived Polio Virus (VDPV) in certain regions of the world has resulted in WHO maintaining its long-standing PHEIC for polio. Successful completion of the EUL process would facilitate the use of nOPV2 in field campaigns to control VDPV events once an outbreak has been identified. Use of the EUL process would allow this novel vaccine to be used prior to WHO Prequalification, enabling an improved vaccine to be utilized in this emergency in advance of obtaining all the data required for traditional product licensure pathway. During this use, data will be gathered to support the traditional WHO Prequalification process and national product licensure pathways.

While the GMP requirements are generally the same as required for the WHO Prequalification program, the efficacy of a vaccine, for example, may be demonstrated by pre-clinical efficacy data in a suitable animal model, alongside clinical immunogenicity that is reasonably predictive of human clinical efficacy. A plan to monitor safety and efficacy in the field must be included, and an EUL is granted initially for 12 months. The manufacturer’s history of successfully prequalifying products may contribute to the decision—especially if specific manufacturing site inspection is difficult to obtain in a timely manner.

Specific to vaccines, though the WHO has provided the EUL, at least one qualified NRA is responsible for providing “oversight of batch release and other post-EUL product safety and manufacturing quality assurance requirements^44^.” This is usually the agency in the manufacturing country. Some countries where epidemics occur are not equipped to do this for certain vaccines. To mitigate this, national and regional regulatory agencies generally should engage with their global regulatory counterparts and WHO Prequalification in a collaborative approach to product assessment and oversight under emergency circumstances, especially when novel outbreak etiologies and novel therapeutic and prophylactic modalities are being proffered.

## Risks and opportunities of these pathways

### When clinical trials have been conducted

Routinely, there is little motivation for manufacturers to produce initial quantities of product in excess of what is required for the clinical trials program. However, in a public health emergency, manufacturers may be called upon to supply larger quantities of product much more quickly. This may deplete clinical trial supplies. If adequate forethought about production has not been undertaken, there may only be enough product for early clinical studies: this results in significant delay to commencement of later phase trials or early larger scale use of the product under one of these pathways, especially during an epidemic (Fig. 1). Mitigation may involve rapid and significant investment of resources in manufacturing, at risk, prior to clinical proof of concept in order to be able to meet demand if the early data support wider use of the product in emergency situations.

### When clinical trials have not been conducted

A key element to utilizing the Animal Rule and analogous facilitated programs is the need for a well-validated animal model. A well-validated animal model may not always exist for “Disease X” and establishing a well-validated animal model can be challenging. In addition, these pathways that utilize animal surrogates may not be applicable to most outbreak scenarios involving new pathogens. Once an outbreak is underway it typically would be feasible and ethical to conduct clinical trials at which point authorization under the animal rule becomes less relevant. Conversely, after an outbreak ends, the opportunity to evaluate efficacy in humans also ends, but at that point the level of urgency also decreases. For novel pathogens, these approaches remain best reserved for instances where probability of human efficacy is higher and apparent at an earlier stage. Without this, the chief value of this approach may be prior to an outbreak or after the outbreak has ended with the aim of supporting use rapidly in a potential future outbreak.

The advantage of many of these pathways is that demand for a product with reasonable presumption of efficacy and safety may be met earlier during a public health emergency, than in a traditional product development and marketing application assessment timeline. In these situations, specific use under emergency authorization may include use in first responders, use in those infected, use in ring programs, and/or use in mass distribution as the situation warrants and as the caveats of the specific emergency use authorization dictate. Manufacturers must commit to collecting further clinical efficacy and safety data, often including clinical trials where feasible and ethical, in conjunction with this emergency use-based field use. Depending on the scientific robustness with which such data are collected and analyzed, these data may provide primary and/or supplementary data for later licensure.

A key downside of these approaches is the data may not help differentiate the potential clinical safety risks of the product from the underlying clinical complications of the disease. Uncontrolled use may confound the long-term safety and efficacy assessments of a product due to the high morbidity and mortality rates during these emergencies. The studies may also be confounded by other comorbidities and other factors typical of the geography, concomitant use of other interventions, or availability (or lack thereof) of other healthcare and medical supplies.

Mitigation of these challenges involves early discussions with regulators. This ensures that there is a common understanding about the natural history of the disease (where possible) and thus a way to try to differentiate drug risk from disease risk and differentiate product efficacy from disease natural history. In addition, where possible, these candidates should be deployed in a controlled clinical study to ensure that the efficacy and safety of the product is appropriately evaluated^45^.

Despite their challenges, these pathways can be used to potentially expedite medical countermeasure availability in a public health emergency for candidates with positive pre-clinical efficacy signals.

## Facilitated regulatory approaches impacting lower income countries

While the abovementioned regulatory agencies have instituted various programs to help expedite the development and assessment of products for use during public health emergencies, many low, and lower-middle income countries have nascent and under-resourced regulatory agencies. For products manufactured in, or used in, countries that cannot assure quality standards, WHO Prequalification is a system developed to help procurers of Prequalification eligible products determine if the products they are procuring meets international regulatory standards for product efficacy, safety and manufacturing quality. Many low-income countries rely on Prequalification listing and the assessment and inspections documents WHO provides them to inform their own national regulatory decision on that specific version of the product. This is done through the WHO-NRA Collaborative Process. WHO cannot “authorize” a product; rather, it “lists” the versions of the products when the WHO assessment determines that the clinical and manufacturing data meet international standards. Like routine product authorizations, routine WHO Prequalification is generally not used in public health emergencies.

Several early engagement opportunities and facilitated accelerated pathways exist when one is focusing on regulators in low-income countries. These national agencies are engaging more with their global regulatory counterparts and WHO Prequalification program staff in a collaborative approach to assess and authorize products (both clinical trials applications and marketing applications) under emergency circumstances, especially when novel outbreak etiologies and novel therapeutic and prophylactic modalities are being proffered.

WHO has instituted a number of pre-emergency activities, described in the latest version of the EUL process^43^. The pre-emergency activities involve establishment of platforms for collaborations between WHO, subject matter experts, NRAs with special expertize, and NRAs where the products will be used (where they differ). WHO establishes expert advisory committees to support each stage of the EUL. In addition, these platforms are used for pre-submission meetings/activities, selection of products, and assessment of submitted data. These activities allow for accelerated decision making upon declaration of a PHEIC or other covered public health emergency. A key benefit of these activities is that the NRAs are involved at early phases of product development and participate in the assessment process. Together, all NRAs and WHO can align on appropriate non-clinical model and clinical study design. This allows for Phase 2b and 3 trials to commence quickly and with the appropriate assistance levels. This can clarify clinical trial endpoints that would be supportive for EUL by WHO and in the target countries.

An additional opportunity for clinical trial discussions are regional health agreements in Sub-Saharan Africa^46–48^, South America^49^, and Asia^50^: these facilitate work-sharing and joint decision-making. For example, the Africa Vaccine Regulatory Forum (AVAREF)^51^ is a continental platform of regulators sponsored by WHO AFRO and WHO-Geneva which coordinates the joint regulatory and ethics board assessments of multinational clinical trials in Africa. Innovator engagement with AVAREF, for example, allows the benefit of joint scientific advice and clinical trial assessment meetings. In addition, AVAREF has established an emergency clinical trial assessment process for multi-country clinical trials. Originally designed only for vaccine trials, AVAREF, despite its anagram, is also available for use with multinational clinical trials application assessment for medicines in Africa.

Outside of the WHO EUL, two other pathways exist to specifically support lower income countries with facilitated assessment, particularly of novel products. They both bring the resources of an agency with specific expertize and WHO to conduct scientific assessments of clinical development programs and marketing authorization applications with opportunities for engagement in the discussions by NRAs from the countries where the product will most likely be used. The use of such pathways brings low income country regulators into the development and assessment processes as a partner so that use of the outputs of the process can be utilized more readily by NRAs where the product will ultimately be used.

*Article 58 of European Commission’s Regulation No 726/2004* is a specific framework, in collaboration with WHO, designed to support lower income countries regarding products to be marketed outside of the European Union^52^. The EMA will assess products of major public health interest, collaborating with the WHO and the NRAs in the countries of use. Regulators, experts and observers from lower income countries participate in the scientific review of the product—both during the development phase and during the marketing application phase. The EMA publishes a scientific opinion regarding the marketing application. This allows under-resourced NRAs to make a decision that leverages the EMA’s assessment (including good clinical and manufacturing practice inspections) and their engagement with EMA and the WHO during the development and marketing application assessments. Recently, an option has been provided for total or partial fee waivers for the manufacturer^53^. While not marketed in the EU, the malaria vaccine RTS,S/AS01 received a positive scientific opinion under Article 58 following trials in seven African countries^54^.

Likewise, in a special program to support access to innovative products in low-income countries, Swissmedic deploys the *Marketing Authorization for Global Health Products (MAGHP)* program. It performs similarly to the EMA’s Article 58 regarding assessment of product development packages and marketing authorization applications in conjunction with WHO and the NRAs from the countries where the product will be used. The difference is that the product, if the assessment is positive, will receive a Swiss marketing authorization even if it will not be used in Switzerland. In the EU, as the product is not intended for use in the EU, the result of the process is a positive opinion, but not a European marketing authorization.

Both of these programs aspire to facilitate a reduction in timelines for development and authorization of products intended solely or primarily for use in lower income countries, thus making needed medicines available faster^55^.

In the near future, regulatory agencies in lower income countries, that have not yet done so, must also become equipped to undertake post-EUL oversight and vigilance surveillance requirements. In addition, they must develop local frameworks to allow emergency use authorization of products during public health emergencies, and that allow the use of candidates that may lack human efficacy data but have both recognized animal efficacy data and initial human safety data.

## Summary and conclusions

Accelerating availability of effective, safe, quality products is essential in a public health emergency. Depending on the context, the feasibility of clinical trials, the strength of animal or clinical surrogate data, and the initial safety profile of the product, one facilitated pathway may be pursued over another or several of these pathways may be pursued simultaneously or sequentially. In these situations, regulators are generally quite willing to discuss putative development plans and regulatory pathways with product developers. Developers should take advantage of such opportunities: these are key to accelerated product development, marketing authorization assessment, and patient access under these facilitated pathways. Generally, this is an iterative process, with decisions being made and modified as further data regarding the emergency and product become available.

Meeting demand via these pathways in the case of a large public health emergency will require robust pre-clinical studies and significant at-risk investment in scaling manufacturing ahead of clinical proof of concept. Because of the rapidly changing nature of public health emergencies, and the requirement for a well-validated animal model, certain pathways may not be able to be utilized in a public health emergency. Historically, most regulatory pathways used in public health emergencies rely on some human efficacy data. Pathways that bring together the manufacturer, NRAs where the product is going to be used, NRAs with specific needed expertize, WHO, and regulatory and clinical experts will accelerate the availability of needed novel medical countermeasures. The benefits of such rapid development could have major impacts, both in terms of lives saved and reduction in disease spread and intensity.

## Supplementary information

Supplementary Note 1
