# Supplementary material for: Navigating facilitated regulatory pathways during a disease X pandemic
Source: NPJ Vaccines. 2020 Oct 23;5:101. doi: 10.1038/s41541-020-00249-5 (PMC7584587; doi:10.1038/s41541-020-00249-5)
Supplement: Supplementary file 1 — Supplementary Note 1 [file 41541_2020_249_MOESM1_ESM.pdf]

Supplementary Note 1
Facilitated Regulatory Pathways & Regional Regulatory Agencies

To date we have identified over 55 facilitated regulatory pathways, from 24 countries or regions. Additionally, we list several regional regulatory initiatives with joint or collaborative procedures. These regional groups offer a centralized regulatory procedure, where a single authorization will be valid in all countries within the regional group. This resource is by no means exhaustive and is current at the time of writing.

Europe and Central Asia

Country/ Region	Facilitated Pathway	Resource
**European Union**	Priority Medicines Scheme (PRIME)	https://www.ema.europa.eu/en/human-regulatory/research-development/prime-priority-medicines
	Accelerated Assessment	https://www.ema.europa.eu/en/human-regulatory/marketing-authorisation/accelerated-assessment
	Conditional Approval	https://www.ema.europa.eu/en/human-regulatory/marketing-authorisation/conditional-marketing-authorisation
	Exceptional Circumstances	https://www.ema.europa.eu/en/human-regulatory/marketing-authorisation/pre-authorisation-guidance
**Russia**	Decree of the Government of the Russian Federation No. 441 of April 3, 2020	http://government.ru/docs/all/127139/
**Turkey**	Human Medicinal Products Priority Assessment	https://www.titck.gov.tr/mevzuat/guideline-for-working-principles-and-procedures-of-human-medicinal-products-priority-assessment-commission-27122018173049
**Ukraine**	Priority Review	http://dls.gov.ua/en/state-service-of-ukraine-on-medicines-and-drugs-control/regulation/
**Switzerland**	Fast-track	https://www.swissmedic.ch/swissmedic/en/home/humanarzneimittel/authorisations/information/anpassung_wl_beschleunigtes_zulassungsverfahren.html
	Authorization human medicine under Art. 13 ATP	https://www.swissmedic.ch/swissmedic/home.webcode.html?webcode=ZL000_00_004e_WL

Latin America & The Caribbean

Country	Facilitated Pathway	Resource
**Brazil**	Priority review Pathway	http://portal.anvisa.gov.br/documents/10181/2718376/RDC_204_2017_.pdf/b2d4ae64-2d91-44e9-ad67-b883c752c094
**Brazil**	Special Procedure for Rare Diseases	http://portal.anvisa.gov.br/documents/10181/2718376/RDC_205_2017_.pdf/996fc46e-216b-44ab-b8c8-2778151b786e
**Chile**	Priority Review	http://www.leychile.cl/Navegar?idNorma=1026879&idVersion=
**Mexico**	Declaration Regarding Public Health Emergencies	http://dof.gob.mx/nota_detalle.php?codigo=5192586&fecha=01/06/2011

Regional Initiative	Resource
Pan American Network for Drug Regulatory Harmonization	www.paho.org/hq/index.php?option=com_content&view=article&id=11825:rede-parf-miembros&Itemid=41777&lang=en
Caribbean Regulatory System	https://carpha.org/What-We-Do/CRS/Caribbean-Regulatory-System

Middle East, North Africa, & Sub-Saharan Africa

Country	Facilitated Pathway	Resource
**Israel**	Fast-track procedure	https://www.health.gov.il/English/MinistryUnits/HealthDivision/MedicalTechnologies/Drugs/Pages/default.aspx
**Kenya**	Fast Tracked Registration	https://pharmacyboardkenya.org/files/?file=drug_reg_guidelines.pdf
**South Africa**	Expedited Review	https://www.sahpra.org.za/
	Abbreviated review process	
**Saudi Arabia**	Priority Review	https://www.sfda.gov.sa/en/drug/resources/Pages/default.aspx
**United Arab Emirates**	Fast Track	https://www.mohap.gov.ae/en/MediaCenter/News/Pages/1950.aspx

Regional Initiative	Resource
African Regional Harmonization Initiative	www.nepad.org/programme/african-medicines-regulatory-harmonisation-amrh
African Vaccines Regulatory Agency	www.afro.who.int/health-topics/immunization/avaref
West African Health Organization	www.wahooas.org/web-ooas/en/actualites/cote-divoire/harmonization-medicines-registration-ecowas-region
East African Commission Medicines Regulation Harmonization	http://mrh.eac.int/eac/
Southern African Development Community Medicines Regulatory Harmonization Project	https://www.sadc.int/themes/health/pharmaceuticals/
Intergovernmental Authority on Development	https://igad.int/divisions/health-and-social-development/1920-igad-and-who-train-for-harmonisation-of-medicines-registration

North America

Country	Facilitated Pathway	Resource
**Canada**	Priority Review	https://www.canada.ca/en/health-canada/services/drugs-health-products/drug-products/applications-submissions/guidance-documents/priority-review/drug-submissions.html
	Notice of Compliance w/Conditions	https://www.canada.ca/en/health-canada/services/drugs-health-products/drug-products/applications-submissions/guidance-documents/notice-compliance-conditions.html
	Extraordinary use of New Drug	https://www.canada.ca/en/health-canada/services/drugs-health-products/biologics-radiopharmaceuticals-genetic-therapies/applications-submissions/guidance-documents/submission-information-requirements-extraordinary-drugs-eunds.html
**United States of America**	Breakthrough Therapy Designation	https://www.fda.gov/patients/fast-track-breakthrough-therapy-accelerated-approval-priority-review/breakthrough-therapy
	Fast-Track Designation	https://www.fda.gov/patients/fast-track-breakthrough-therapy-accelerated-approval-priority-review/fast-track
	Accelerated Approval	https://www.fda.gov/patients/fast-track-breakthrough-therapy-accelerated-approval-priority-review/accelerated-approval
	Priority Review	https://www.fda.gov/patients/fast-track-breakthrough-therapy-accelerated-approval-priority-review/priority-review
	Limited Population Pathway	https://www.fda.gov/drugs/development-resources/limited-population-pathway-antibacterial-and-antifungal-drugs-lpad-pathway
	Regenerative Advanced Therapy Designation	https://www.fda.gov/vaccines-blood-biologics/cellular-gene-therapy-products/regenerative-medicine-advanced-therapy-designation

	Tropical Disease Priority Review Voucher Program	https://www.fda.gov/about-fda/center-drug-evaluation-and-research-cder/tropical-disease-priority-review-voucher-program
	Animal Rule	www.fda.gov/regulatory-information/search-fda-guidance-documents/product-development-under-animal-rule

South Asia, East Asia & Pacific

Country	Facilitated Pathway	Resource
Australia	Priority Determination	https://www.tga.gov.au/publication/priority-determination
	Provisional Approval Pathway	https://www.tga.gov.au/provisional-approval-pathway-prescription-medicines
China	Priority review	http://www.nmpa.gov.cn/WS04/CL2196/324193.html
	Conditional Approval for the marketing of medical devices	http://subsites.chinadaily.com.cn/nmpa/2019-12/20/c_456289.htm
	Special review for innovative drug registration	http://www.ccpie.org/news/download/2015pharm-13.pdf
Japan	Priority Review	https://www.pmda.go.jp/english/review-services/reviews/0001.html
	Conditional and Term-Limited Approval	https://www.pmda.go.jp/review-services/drug-reviews/0045.html
	Conditional Early Approval	https://www.pmda.go.jp/review-services/drug-reviews/0045.html
	Sakigake	https://www.mhlw.go.jp/english/policy/health-medical/pharmaceuticals/140729-01.html
Indonesia	Accelerated approval system (100 WD)	https://www.pom.go.id/new/view/direct/pedoman
South Korea	Priority Review	https://www.mfds.go.kr/eng/wpge/m_17/de011008l001.do
	Fast Track Review	
	Rolling Submission	
	Conditional Approval with Phase 2 Results	
Singapore	Priority Review	https://www.hsa.gov.sg/therapeutic-products/register/guides/new-drug/abridged-evaluation
Singapore	Special Access Route	https://www.hsa.gov.sg/therapeutic-products/register/special-access-routes/import-for-patients
Taiwan	Priority Review	https://www3.cde.org.tw/eng/faq/faq_more?id=1453
	New drug priority review	
	Bridging Study Evaluation	
	Accelerate Approval	
Thailand	Priority Review	http://www.fda.moph.go.th/sites/Drug/EN/Pages/Main.aspx
Vietnam	Priority Approval	https://dav.gov.vn/van-ban-quan-ly/legal-normative-documents-vbe40.html

Regional Initiative	Resource
Asian Pacific Economic Community	https://www.apec.org/Groups/Committee-on-Trade-and-Investment/Life-Sciences-Innovation-Forum
Association of Southeast Asian Nations Regulatory Harmonization	https://asean.org/storage/2017/09/ASEAN-Principles-HRR-endorsed-SEOM-350-July-2019.pdf
